# A Tale of Chivalry

**Published:** 1890-06-28

**Authors:** 


### The Hospital

A Weekly Journal of

**Science, Medicine, Nursing, and Philanthropy.**

*For Hospitals, Asylums, Medical Practitioners, Students, Nurses, Families, Parishes, Congregations, and the Charitable Public.*

Vol. VIII.—No. 196.] [June 28, 1890.

### A Tale of Chivalry.

In the winter of 1866, twenty-three years ago, a young medical student of the London Hospital found himself among the waifs and strays in a ragged school at the East-end of London. On the particular evening in question he came face to face with one unhappy street boy whose simple story was so pathetic and whose case was so desperate that the student there and then took charge of the lad, and made himself responsible for his future support and well-being. But this boy's story, although it concerned principally himself, incidentally revealed so much suffering and degradation among innumerable other boys of his own class that his listener was moved to the depths of his soul. He began thereupon to search in the places which his little homeless waif had been accustomed to haunt, and where he had found all that he knew of home. The student, searching earnestly, discovered large numbers of mere children, not only in the East-end, but in the Centre, South, North, and even West of London, who had no other home whatever but the common lodging-house. Those children, if they were unable during the day to pick up the few halfpence necessary to secure a bed in a lodging-house, were obliged to walk the streets all night or to find precarious shelter where they could. As a matter of fact, some of them slept in the doorways of private houses, some under archways, some in deserted markets, some in waggons and carts, some by the river side, and many in alleys and courts all over the metropolis.

This was a revelation to the youthful medical student; but it was an apocalypse whose mystery he had laid bare for himself. Having seen with his own eyes he could not sit down and do nothing as if he had not seen. On his own responsibility alone he opened a very small house in Stepney Causeway, and here he soon had a numerous family of waifs and strays, sought for and rescued by his own hand from the homelessness, destitution, and degradation of the London streets.

Such was the beginning of what is now the largest Orphanage in the world. The building up of a professional reputation, of a business, of a public association, is often a slow, and always a laborious process. Things that are to endure are not fashioned in a day. Even if a man have unlimited money he may easily find himself a failure if he attempts to conduct an orphanage. Dr. Barnardo, for it is of him we speak, had, as we gather, no money at all of his own when he resolved, in the spirit of a hero, to give up what professional prospects he might have, and to devote his life to the ragged, the outcast, the potential criminal, and the hopeless of the London slums. But he began his work with such resources as he possessed; with the qualification first of an overwhelming pity for the boys, of a resolution to do all he could, whether much or little, and of a strong and persevering energy which was quite as remarkable as that of a Stanley, a Wellington, or a Captain Cook.

Carlyle gives us a practical standard whereby we may prove of what sterling value a man is. What has he done? That is the question. Not what has he said, or what has he purposed. But what has he, of his own initiative and by his own resolution and persistent energy, carried to a completed result? This is the best and the final standard of a man's capacity and worth. Dr. Barnardo, as we have seen, had the courage to found an Orphanage when he was merely a young medical student. In course of time, though by no means quickly, his one house became two; and the boys, who were at first gathered in from the East-end slums only, began to be sent from other parts of London, and even from the country. Lost waifs themselves gradually came to know of the home, and when they found destitution no longer tolerable, would apply of their own accord. As the work grew friends increased, and it became at length possible to offer to all destitute street boys a home and a parent.

Those kind-hearted persons who are grateful when they find willing and able hands to carry out their own benevolent wishes soon began to understand the character of the youthful doctor. He had proved himself signally successful with boys: why should he not open a home for girls also? The appeal was urgent, and the Ilford Homes for Girls, which only those who have seen can really know the value of, began to arise. The writer has been familiar with those cottage homes for years. Each one has its Christian mother, its clean rooms, its comfortable beds, its neat garden, and its full complement of wholesome and merry children. Who shall estimate the happiness those children now enjoy, or the misery and destitution from which they have been saved?

The practical capacity of Dr. Barnardo for dealing with large numbers of children of both sexes and for acting as general of the forces to those who are set over them is altogether admirable. The doctor has a miniature kingdom under his charge. His 3,000 orphans are of all ages and both sexes. Some of them require to be nursed; some need teachers; some trade masters and mistresses; some drill sergeants; some emigration superintendents. The trades taught are almost all that a large colony would need. They include shoemaking, tailoring, carpentering, brush-making, box-making, wood-chopping, and so on. The institution has a church and a hospital, a temperance society and a babies' castle. It would be difficult indeed to say what it has not got. Connected with it are no fewer than thirty-eight subordinate and separate institutions. Merely to name some of these, shows how comprehensive are Dr. Barnardo's plans, and how infinitely varied is his work. There is, for example, a farm school at Buckenhill, in Worcestershire, given rent free by Mr. R. Phipps, J.P., and as a complement to this an Industrial Farm of 10,000 acres at Birtle, in Manitoba. There is the Babies' Castle for Infants at Hawkhurst, in Kent, and a Convalescent Seaside Home at Felixstowe, in Suffolk. There is the Union Jack Shoe-Black Brigade at Limehouse, and the Open-all-night Shelter for Homeless Boys and Girls at Stepney Causeway; there is the Rescue Home for young girls in special danger, with a private address, and the Children's Fold for Little Cripples in Victoria Park; there is the Missionary Church, which accommodates over 3,000 people, and which is full every Sunday, and there is Her Majesty's Hospital for sick children, which contains seventy beds. All this is a sufficiently wonderful history of work started by a mere medical student, and accomplished in twenty-three years.

The briefest possible account of Dr. Barnardo's money matters is all we can give. On an average he has over 3,000 mouths to feed and 3,000 backs to clothe every day. Besides this he has to pay teachers, tradesmen of all kinds, superintendents, matrons, and other helpers. His average expenditure is £150 a day, and his income from endowments is *nil.* For food alone he spends £100 a day. Over against this enormous expenditure may be placed the gifts of last year. The amount received was the largest ever contributed in a single twelve months, and reached the extraordinary total of £106,722. A moment's glance at the subscription list shows that this sum was given by nearly 80,000 different persons, and by far the larger proportion of these gave less than £1 each. There were not 2,000 subscribers whose subscriptions exceeded £10.

Some persons object against Dr. Barnardo that he is a determined Protestant. But why is he a determined Protestant? Our article has been written to little purpose if readers cannot answer that question for themselves. Dr. Barnardo is a determined Protestant because he is a determined man. What would he have been without determination of character, and where would his Orphanage have been? For our part, we consider his ecclesiastical position to be a matter of no moment whatever, compared with the value of the splendid work he has done. Who questioned whether Wellington was a Protestant or a Catholic when he was fighting our battles on the peninsula? Who cares whether Stanley is orthodox or heterodox so long as his morals are sound and his practical work good? The Marquis of Lorne, presiding at the Anniversary meeting of the Orphanage the other day, bore emphatic testimony to the great and enduring work which he himself had been a constant witness of during the five years of his connection with the institution. Such trusted philanthropists as Canon Fleming, Sir S. A. Blackwood, and many others, stood shoulder to shoulder with the Marquis of Lorne in their resolute support of the doctor and his deeds of philanthropic valour. One of the surest of all evidences of a man's powerful personality is a host of enemies. Dr. Barnardo has enough of these, and more than enough; but of this he and all others may be certain, that so long as he carries on his great work with his present singleness of eye and excellence of method and result, all the enemies in the world cannot defeat his purpose or stay his hand.

